# Screening for Cervical Cancer Precursors With p16/Ki-67 Dual-Stained Cytology: Results of the PALMS Study

**DOI:** 10.1093/jnci/djt235

**Published:** 2013-10-04

**Authors:** Hans Ikenberg, Christine Bergeron, Dietmar Schmidt, Henrik Griesser, Francisco Alameda, Claudio Angeloni, Johannes Bogers, Roger Dachez, Karin Denton, Jalil Hariri, Thomas Keller, Magnus von Knebel Doeberitz, Heinrich H. Neumann, Luis M. Puig-Tintore, Mario Sideri, Susanne Rehm, Ruediger Ridder, for the PALMS Study Group

**Affiliations:** **Affiliations of authors:**Cytomol, Frankfurt, Germany (HI); Laboratoire Cerba, Cergy Pontoise, France (CB); Institute for Pathology, Mannheim, Germany (DS); Center for Pathology and Cytodiagnostics, Cologne, Germany (HG); Hospital del Mar, Barcelona, Spain (FA); Unità Gestionale Screening Regionale, Ospedale Atri, Italy (CA); Labo Lokeren - Campus Riatol, Antwerp, Belgium (JB); Institute Alfred Fournier, Paris, France (RD); North Bristol NHS Trust, Bristol, United Kingdom (KD); Sønderborg Hospital, Sønderborg, Denmark (JH); Acomed statistik, Leipzig, Germany (TK); Institute for Pathology, University of Heidelberg, Heidelberg, Germany (MvkD); Institute for Pathology, Nordhorn, Germany (HHN); University of Barcelona, Barcelona, Spain (LMP-T); European Institute of Oncology, Milan, Italy (MS); Roche mtm laboratories, Mannheim, Germany (SR, RR).

## Abstract

**Background:**

Pap cytology is known to be more specific but less sensitive than testing for human papillomavirus (HPV) for the detection of high-grade cervical intraepithelial neoplasia (CIN2+). We assessed whether p16/Ki-67 dual-stained cytology, a biomarker combination indicative of transforming HPV infections, can provide high sensitivity for CIN2+ in screening while maintaining high specificity. Results were compared with Pap cytology and HPV testing.

**Methods:**

A total of 27349 women 18 years or older attending routine cervical cancer screening were prospectively enrolled in five European countries. Pap cytology, p16/Ki-67 immunostaining, and HPV testing were performed on all women. Positive test results triggered colposcopy referral, except for women younger than 30 years with only positive HPV test results. Presence of CIN2+ on adjudicated histology was used as the reference standard. Two-sided bias-corrected McNemar *P* values were determined.

**Results:**

The p16/Ki-67 dual-stained cytology positivity rates were comparable with the prevalence of abnormal Pap cytology results and less than 50% of the positivity rates observed for HPV testing. In women of all ages, dual-stained cytology was more sensitive than Pap cytology (86.7% vs 68.5%; *P* < .001) for detecting CIN2+, with comparable specificity (95.2% vs 95.4%; *P* = .15). The relative performance of the tests was similar in both groups of women: younger than age 30 and 30 years or older. HPV testing in women 30 years or older was more sensitive than dual-stained cytology (93.3% vs 84.7%; *P* = .03) but less specific (93.0% vs 96.2%; *P* < .001).

**Conclusions:**

The p16/Ki-67 dual-stained cytology combines superior sensitivity and noninferior specificity over Pap cytology for detecting CIN2+. It suggests a potential role of dual-stained cytology in screening, especially in younger women where HPV testing has its limitations.

## Introduction

Sensitivity of a single cytology test for the detection of cervical intraepithelial neoplasia of grade 2 or higher (CIN2+, or high-grade CIN, HGCIN) is unsatisfactorily low (1,2). To improve sensitivity, testing for the presence of high-risk human papillomavirus has been proposed as an alternative or adjunct tool for cervical cancer screening. Numerous studies have shown that HPV testing provides high sensitivity for CIN2+ (2–8). However, specificity of screening women for HGCIN with HPV testing is limited because most HPV infections are transient, and only a low proportion persists and may progress into transforming infections and HGCIN (9). Due to the high prevalence of HPV infections in younger women, HPV testing currently is not recommended for screening women younger than age 30 (10).

Detection of overexpression of p16^INK4a^ (p16), a biomarker of transforming HPV infections and precancerous cervical lesions, has been shown to be an efficient tool in managing patients with atypical squamous cells of undetermined significance (ASC-US) or low-grade squamous intraepithelial lesion (LSIL) cytology results (11–14), and for triaging HPV-positive women (15,16). Most recently, several studies have analyzed the diagnostic performance of a novel p16/Ki-67 dual-stained cytology approach that combines simultaneous p16 and Ki-67 detection in the same cell as a hallmark of cell-cycle deregulation (17–22). In normal cells, the expression of p16 and Ki-67 is mutually exclusive. p16/Ki-67 dual-stained cytology, a morphology independent test, showed similar sensitivity and significantly higher specificity than HPV testing in ASC-US or LSIL triage (17), and in women with negative Pap cytology but positive HPV screening results (18).

Here we investigated the p16/Ki-67 dual-stained cytology approach in a large prospective diagnostic screening study, the Primary ASC-US and LSIL Marker Study (PALMS). This pan- European study was performed to estimate the sensitivity and specificity of the p16/Ki-67 dual-stained cytology and to compare it with Pap cytology testing in a routine European cervical cancer screening population of women 18 years or older, and to HPV testing in women 30 years or older. The diagnostic performance of this novel biomarker approach for triaging ASC-US and LSIL as well as HPV-positive screening results in the PALMS study are reported separately.

## Subjects and Methods

### Participants and Procedures

Women18 years or older undergoing routine cytology-based cervical cancer screening were enrolled in gynecologist practices and hospital-based screening centers (n = 196) in Belgium, France, Germany, Italy, and Spain. Exclusion criteria included pregnancy and previous hysterectomy. Written informed consent was obtained from all participating subjects. The study was approved by central and local ethics committees and conducted in accordance with good clinical practice guidelines and the Declaration of Helsinki (study registered under DRKS00000408 at http://apps.who.int/trialsearch/).

All women received Pap cytology, p16/Ki-67 dual-stained cytology, and HPV testing. Either conventional Pap cytology or liquid-based cytology (LBC; ThinPrep Pap Test, Hologic, Marlborough, MA; or, SurePath, BD Diagnostics, Burlington, NC) was used. During the screening visit, a first cervical sample was collected for Pap cytology testing using broom-type or brush/spatula sampling devices. For conventional cytology, a first slide was prepared for Pap screening, and residual material on the sampling device was used to prepare a second slide for p16/Ki-67 dual-staining (“split sample technique”). For LBC, residual material in the vial was used for p16/Ki-67 dual-staining. A second cervical sample was taken from all study participants using the DNAPAP Cervical Sampler (Qiagen, Hilden, Germany) for high-risk human papillomavirus DNA testing performed in six independent central laboratories in Italy, France, and Germany using the Digene HC2 HPV DNA Test (Qiagen).

All subjects with abnormal Pap cytology results (ASC-US or worse; ASC-US+), and/or a positive p16/Ki-67 dual-stained cytology result, and/or a positive high-risk human papillomavirus test result were referred for colposcopy, unless HPV was the only positive test in women younger than 30 years. Subjects with negative results in all tests completed the study upon receipt of results.

Pap cytology was interpreted in local cytology laboratories (n = 16) using the Bethesda System for reporting cervical cytology (23). Dual-immunostaining for p16/Ki-67 was performed centrally using the CINtec PLUS kit (Roche mtm laboratories, Mannheim, Germany) according to the manufacturer’s instructions and as described previously (see also Supplementary Methods) (17,18). The p16/Ki-67 dual-stained slides were reviewed by individual members of a pool of eight independent cytotechnologists. Each slide was reviewed independently by two cytotechnologists for the presence of p16/Ki-67 dual-stained cells. Cases showing one or more dual-stained cell(s) (ie, a positive dual-stained cytology test result) during cytotechnologist review were confirmed by individual pathologists recruited from a group of five pathologists (DS, FA, JB, KD, JL). All reviewers (cytotechnologists/pathologists) were informed about patient’s age but blinded to all other study results.

Colposcopy was performed according to accepted diagnostic standards and national guidelines in the respective countries. In line with current clinical practice, colposcopists were aware of Pap cytology and HPV test results but blinded to any dual-stained cytology results. Biopsies were taken if clinically indicated. Cases where no biopsies were taken during the colposcopic examination were considered negative for disease. Biopsy-confirmed CIN2+ was used as clinical end point for the study. All local histology results were verified by members of an independent quality control (QC) review board comprising a total of five pathologists, blinded to all study results. The QC review of each histologic diagnosis was assigned to one board member and performed on hematoxylin and eosin (H&E)-stained slides. QC review results concordant with previous local diagnoses were considered final study diagnoses, unless CIN2+ had been diagnosed by the local pathologist. All CIN2+ cases and cases with discrepant results between local pathologists and first QC reviewers were subjected to an extended QC review by at least one additional QC pathologist. Majority agreement results established final H&E diagnoses (reference standard H&E). Cases without a majority agreement diagnosis were adjudicated during a joint review session. H&E results were used as primary reference standard for study analyses.

For all tissue specimens, a parallel section stained for p16 using the CINtec Histology Kit (Roche mtm laboratories) was separately evaluated regarding its staining pattern (ie, diffuse positive vs focal/negative). In cases with diffuse staining, but no CIN diagnosed per final H&E result, and in cases with focal/negative staining pattern, but CIN2+ per final H&E result (combined n = 70 of 785 cases), a separate conjunctive analysis of the H&E and p16 stained slides was performed by three QC review pathologists during a separate adjudication meeting (reference standard H&E *corr*; see Supplementary Tables). The p16 staining interpretation did not overrule morphologic interpretation.

### Statistical Methods

With a reference standard available only for patients with at least one positive test result, data analysis used a verification of only positive tests (VOPT) design (24). Specifically, relative diagnostic measures were analyzed: ratio of true-positive fractions and ratio of false-positive fractions. These relative measures were calculated using dual-stained cytology in the numerator and the other test (Pap, HPV) in the denominator. With three tests applied, probability of disease among triple test negatives is nearly zero, so absolute measures of diagnostic accuracy could be estimated. Most results are reported as absolute measures for ease of interpretation. Both relative and absolute measures are reported with 95% confidence intervals.

Sample size calculation for the comparison of sensitivities of Pap versus dual-stained cytology testing in screening for CIN2+ assumed disease prevalence of 0.8%. Assuming a 75% follow-up compliance rate, more than 25 500 subjects gave 90% power to detect as statistically significant an increase in sensitivity from 60% to 80% (24).

Verification bias correction was performed to correct diagnostic accuracy estimates for disparities in colposcopy follow-up for the various test results (25,26), whereby for each test the combination disease rate was calculated and applied. Two-sided bias-corrected McNemar *P* values were determined with *P* < .05 considered statistically significant (see Supplementary Methods for more details).

## Results

### Enrollment and Demographics

**Figure 1.**
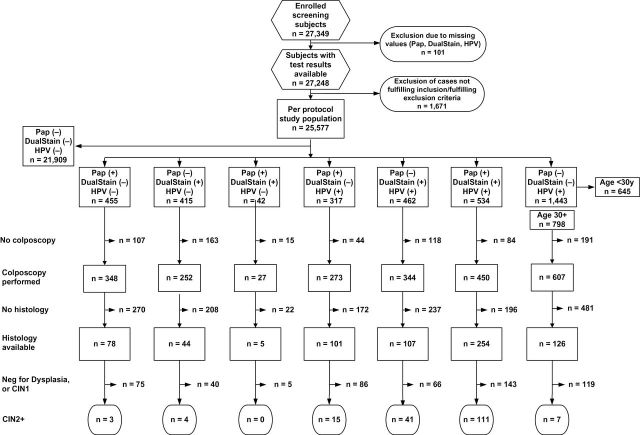
Test results and outcomes. Positive Pap cytology results are defined as atypical squamous cells of undetermined significance (ASC-US) or worse (Pap +). A positive p16/Ki-67 dual-stained cytology (Dual Stain +) is defined as the presence of one or more double- immunoreactive cell(s). A RLU/cut-off value ratio of 1.0 or higher was considered a positive test result (HPV +) for the Digene HC2 HPV DNA Test.

A total of 27 349 women attending routine cervical cancer screening were enrolled into the study: 12 226 (44.7%) in Germany, 5250 (19.2%) in Italy, 4034 (14.8%) in France, 3929 (14.4%) in Spain, and 1910 (7.0%) in Belgium. The mean age of the screening population enrolled into the study was 39.9 years (standard deviation ± 11.7; 18–74). A quarter (6798; 24.9%) of the 27 248 women that had all three tests performed were 18–29 years of age, and 75.1% (20 450) were 30 years or older. Complete data sets were available for 25 577 subjects (Figure 1). A total of 2301 colposcopies were performed, representing 76% (2301 of 3023) of colposcopies required per protocol. There was a disparity in the attendance rate to colposcopy dependent on positive individual test results, with a higher rate of women undergoing colposcopy based on ASC-US+ (81.5%) or positive HPV test (79.3% within the group of women 30 years or older), compared with women positive for p16/Ki-67 dual-stained cytology (73.8%). Estimates of diagnostic accuracy therefore were corrected for verification bias.

### Prevalence of Positive Test Results

Among the 27 248 subjects with all three tests performed, the overall prevalence of positive dual-stained cytology test results was 5.4%, similar to the prevalence of ASC-US+ (5.2%) and half of the prevalence of HPV (10.7%). Positivity rates were generally higher in the group of women 18–29 years of age versus women 30 years or older. Prevalence rates of positive dual-stained cytology were comparable with ASC-US+ and approximately half of the respective HPV prevalence rates in both age groups (Table 1).

**Table 1.** Prevalence of positive test results for Pap cytology, p16/Ki-67 dual-stained cytology, and human papillomavirus testing*

Age group	Pap cytology (ASC-US or worse)		Dual-stained cytology positive		HPV positive	
	No.	(%)	No.	(%)	No.	(%)
Women of all ages (n = 27 248)	1407	(5.2)	1462	(5.4)	2918	(10.7)
Women 18–29 y (n = 6798)	563	(8.3)	605	(8.9)	1376	(20.2)
Women 30–65 y (n = 20 450)	844	(4.1)	857	(4.2)	1542	(7.5)

* ASC-US = atypical squamous cells of undetermined significance; HPV = human papillomavirus. The presence of one or more cell(s) showing simultaneous immunoreactivity for both p16 and Ki-67 was used to define a positive test result for dual-stained cytology.

### Tests Performance Characteristics

The assessments of the diagnostic performance characteristics were based on 181 cases of biopsy-confirmed CIN2+ (including 100 CIN3+), using histopathologic reference standard H&E. Additional adjudication on H&E plus p16 stained slides on a subgroup of 8.9% (70 of 785) of biopsy cases where the histology result was not supported by the histologic p16 staining pattern (reference standard H&E *corr*) revealed a total number of 205 CIN2+ (111 CIN3+).

**Figure 2.**
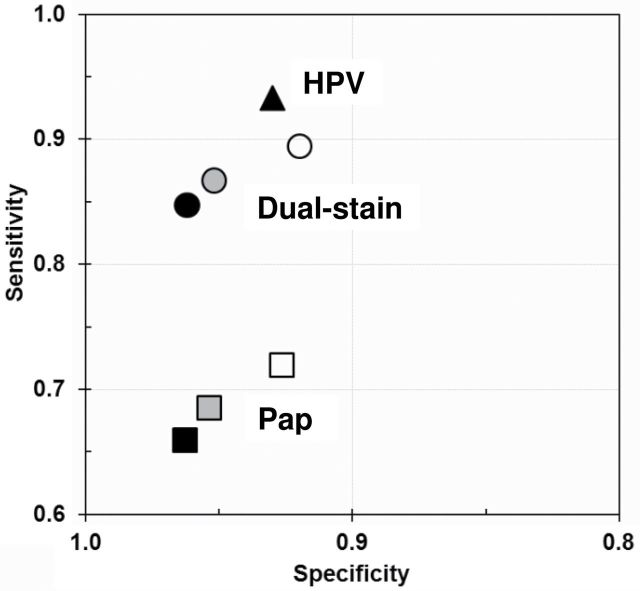
Diagnostic performance for detecting CIN2+ (Reference standard H&E). Receiver operating characteristic graph is shown for Pap cytology (**squares**), dual-stained cytology (**circles**), and HPV (**triangle**) for women 18–65 years of age (**gray fill**), younger than 30 years (**white fill**), and 30 years or older (**black fill**).

Among women of all ages, sensitivity for biopsy-confirmed CIN2+ (reference standard H&E) of dual-stained cytology was 86.7%, statistically significantly higher than Pap cytology (68.5%; ratio of true-positive fractions = 1.265, *P* < .001) (Tables 2 and 3). Specificity rates for both tests were comparable (95.2% vs 95.4%; ratio of false-positive fractions = 1.049; *P* = 0.15). A similar statistically significant effect of increased sensitivity of dual-stained cytology over Pap cytology was observed when analyzing data for women 18–29 years or women 30 years or older (Tables 2 and 3). HPV testing in screening of women 30 years or older was more sensitive for CIN2+ than dual-stained cytology (93.3% vs 84.7%; *P* = .03) but less specific (93.0% vs 96.2%; *P* < .001). Figure 2 summarizes the diagnostic performance as a receiver operating characteristic graph.

Generally, similar absolute and relative diagnostic performance characteristics were observed when alternative reference standard H&E *corr* was used (Supplementary Tables 1 and 2; Supplementary Figure 1). However, sensitivity rates of both dual-stained cytology and HPV testing were found higher when using reference standard H&E *corr*, whereas Pap cytology sensitivity rates were slightly lower or unchanged. In women younger than age 30, dual-stained cytology showed a sensitivity higher than 93% for both CIN2+ and CIN3+ cut-offs, compared with Pap cytology sensitivity rates of 67.7% and 74.4%, respectively (Supplementary Table 1). In women 30 years or older, sensitivity rates of Pap cytology for CIN3+ were unchanged (69.1% vs 69.0%), whereas testing for the morphology-independent biomarkers showed sensitivity rates that were 1.3% (p16/Ki-67: 88.5 vs 87.2%) and 3.8% (HPV: 100 vs 96.2%) higher when using reference standard H&E *corr* (Table 2 and Supplementary Table 1).

**Table 2.** Sensitivity, specificity, and predictive values of Pap cytology, p16/Ki-67 dual-stained cytology, and human papillomavirus testing in screening for CIN2+ and CIN3+*

Subgroup	CIN2+		CIN3+		CIN2+	
	Sensitivity % (95% CI)	Specificity % (95% CI)	Sensitivity % (95% CI)	Specificity % (95% CI)	PPV %	NPV %
Women 18–65 y (n = 25 577; 181 CIN2+, 100 CIN3+)						
Pap cytology	68.5 (61.2 to 75.0)	95.4 (95.1 to 95.6)	73.6 (63.8 to 81.5)	95.1 (94.8 to 95.4)	13.3	99.7
Dual-stained cytology	86.7 (81.1 to 90.9)	95.2 (94.9 to 95.4)	87.4 (79.5 to 92.5)	94.8 (94.5 to 95.1)	15.6	99.9
Women 18–29 y (n = 6372; 70 CIN2+, 37 CIN3+)						
Pap cytology	71.9 (59.8 to 81.5)	92.6 (92.0 to 93.3)	80.9 (63.9 to 91.0)	92.2 (91.5 to 92.8)	14.4	99.5
Dual-stained cytology	89.4 (80.2 to 94.6)	92.0 (91.2 to 92.6)	87.3 (72.8 to 94.6)	91.3 (90.5 to 92.0)	16.1	99.8
Women 30–65 y (n = 19 205; 111 CIN2+, 63 CIN3+)						
Pap cytology	65.9 (56.5 to 74.3)	96.3 (96.0 to 96.5)	69.0 (56.4 to 79.4)	96.1 (95.8 to 96.3)	12.5	99.7
Dual-stained cytology	84.7 (76.8 to 90.3)	96.2 (95.9 to 96.5)	87.2 (76.5 to 93.5)	95.9 (95.6 to 96.2)	15.3	99.9
HPV	93.3 (85.9 to 96.9)	93.0 (92.6 to 93.4)	96.2 (86.0 to 99.0)	92.7 (92.4 to 93.1)	9.3	99.9

* CIN2+ (CIN3+), cervical intraepithelial neoplasia grade 2 (3) or worse; HPV = human papillomavirus; CI = confidence interval; PPV = positive predictive value; NPV = negative predictive value. Data are provided for reference standard H&E.

**Table 3.** Relative performance characteristics of Pap cytology, p16/Ki-67 dual-stained cytology, and human papillomavirus testing*

Subgroup	CIN2+				CIN3+			
	Relative sensitivity rTPF (95% CI)	*P* rTPF	Relative specificity rFPF (95% CI)	*P* rFPF	Relative sensitivity rTPF (95% CI)	*P* rTPF	Relative specificity rFPF (95% CI)	*P* rFPF
Women 18–65 y (n = 25 577; 181 CIN2+, 100 CIN3+)								
Dual-stained cytology vs Pap cytology	1.265 (1.127 to 1.421)	<.001	1.049 (0.983 to 1.120)	.15	1.187 (1.033 to 1.364)	.02	1.069 (1.005 to 1.136)	.03
Women 18–29 y (n = 6372; 70 CIN2+, 37 CIN3+)								
Dual-stained cytology vs Pap cytology	1.243 (1.043 to 1.495)	.02	1.094 (0.990 to 1.208)	.08	1.078 (0.863 to 1.347)	.51	1.119 (1.019 to 1.228)	.02
Women 30–65 y (n = 19 205; 111 CIN2+, 63 CIN3+)								
Dual-stained cytology vs Pap cytology	1.285 (1.105 to 1.494)	.001	1.020 (0.935 to 1.112)	.66	1.263 (1.056 to 1.511)	.01	1.036 (0.955 to 1.125)	.39
Dual-stained cytology vs HPV	0.897 (0.814 to 0.990)	.03	0.548 (0.510 to 0.589)	<.001	0.898 (0.807 to 1.000)	.05	0.563 (0.526 to 0.602)	< .001

* For detection of cervical intraepithelial neoplasia grade 2 or worse (CIN2+) and grade 3 or worse (CIN3+).

HPV = human papillomavirus; CI = 95% confidence intervals; rTPF = ratio of true-positive fractions (relative sensitivity); rFPF = ratio of false-positive fractions (relative 1-specificity).

Data are provided for reference standard H&E. Two-sided bias-corrected McNemar *P* values are reported.

The Pap cytology methods used within this study were conventional Pap smears in 9773 (38.2%) of all cases, ThinPrep Pap Test in 8708 (34.0%) of cases, and SurePath LBC in 7096 (27.7%) of cases. The study was not designed to demonstrate differences in the performance of Pap or dual-stained cytology tests dependent on the cytology method used for slide preparation. However, substantial differences were observed for the performance of Pap cytology dependent on which cytology method was used. Although among all Pap cytology methods the use of ThinPrep LBC specimens revealed the highest sensitivity for CIN2+ (84.7%), specificity was average (Table 4). Substantially lower Pap cytology sensitivity rates for detecting CIN2+ were observed using SurePath LBC (58.5%) or conventional smears (63.5%). Specificity of Pap cytology was highest using conventional smears and lowest in SurePath LBC specimens. In contrast, the variability of dual-stained cytology observed for the different cytology methods was substantially lower. Sensitivity rates for CIN2+ ranged from 91.4% for ThinPrep to 83.9% for SurePath and 85.0% for conventional cytology specimens (Table 4). Using reference standard H&E *corr*, sensitivity rates of dual-stained cytology for CIN2+ ranged from 95.6% for ThinPrep to 88.1% for SurePath, and 87.2% for conventional cytology specimens (Supplementary Table 3).

**Table 4.** Absolute and relative performance characteristics of Pap cytology and p16/Ki-67 dual-stained cytology for detection of cervical intraepithelial neoplasia grade 2 or worse per Pap cytology method*

Method	Dual-stained cytology		Pap cytology		Dual-stained vs Pap cytology	
	Sensitivity % (95% CI)	Specificity % (95% CI)	Sensitivity % (95% CI)	Specificity % (95% CI)	Relative sensitivity rTPF (95% CI)	Relative specificity rFPF (95% CI)
All	86.7 (81.1 to 90.9)	95.2 (94.9 to 95.4)	68.5 (61.2 to 75.0)	95.4 (95.1 to 95.6)	1.265 (1.127 to 1.421)	1.049 (0.983 to 1.120)
Conventional	85.0 (75.4 to 91.3)	95.7 (95.3 to 96.1)	63.5 (52.4 to 73.4)	97.5 (97.1 to 97.8)	1.338 (1.107 to 1.618)	1.707 (1.498 to 1.944)
SurePath	83.9 (71.0 to 91.8)	94.9 (94.4 to 95.4)	58.5 (43.6 to 72.0)	93.0 (92.3 to 93.5)	1.435 (1.071 to 1.921)	0.722 (0.644 to 0.810)
ThinPrep	91.4 (80.9 to 96.4)	94.8 (94.3 to 95.2)	84.7 (72.5 to 92.1)	95.0 (94.6 to 95.5)	1.079 (0.941 to 1.237)	1.053 (0.947 to 1.170)

* CIN2+ = cervical intraepithelial neoplasia grade 2 or worse; CI = confidence interval; rTPF = ratio of true-positive fractions (relative sensitivity); rFPF = ratio of false-positive fractions (relative 1-specificity). Reference standard hematoxylin and Eosin (H&E).

Similar to the observation for the sensitivity estimates, the variability in specificity obtained for the various Pap methods was widely eliminated when using the dual-stain protocol and interpretation algorithm on the same specimens that have been used for establishing the Pap result (Table 4; Supplementary Tables 4–6).

## Discussion

The PALMS study data show that dual-stained cytology may provide both high sensitivity and specificity for detecting HGCIN in a single test. Dual-stained cytology increased the sensitivity for CIN2+ by 18% over Pap cytology (*P* < .001) in women of all ages, with a specificity of 95.2% (Table 2). Furthermore, sensitivity of dual-stained cytology is reaching more than 90% of the sensitivity level of HPV testing in women 30 years or older, with a relatively small but statistically significant difference. At the same time, specificity of dual-stained cytology is significantly higher as compared with HPV testing, reducing the number of false-positive screening test results by almost 50%.

This is the first study evaluating the utility of an immunocytochemical staining approach based on the p16 biomarker in screening for HGCIN. Previous studies were limited to the triage of abnormal Pap cytology (14,17,19–22) or positive HPV test results (15,16,18). Initial studies were also predominantly based on p16 single-stained cytology protocols combined with the morphologic interpretation of immunoreactive cells (14).

In this study, there was no complete disease ascertainment for all three tests because women younger than 30 years and testing positive only for HPV were not referred to colposcopy. Although this is in concordance with clinical practice where HPV testing is not recommended for co-testing in screening of younger women (10), this represents a weakness of the study, in addition to its generally rather low rate of biopsies sampled during colposcopic procedures. Local ethical review boards in Europe were reluctant to accept a more stringent colposcopy and biopsy sampling protocol. Therefore, only assumptions can be made when trying to understand the performance of dual-stained cytology in women younger than 30 years compared with HPV testing.

Assuming that the rate of potential high-grade disease missed by both dual-stained cytology and Pap cytology in younger women may be rather small and thus the overall sensitivity of dual-stained cytology in younger women may be at least comparably high as in women 30 years or older, dual-stained cytology may be of particular interest as a screening adjunct or primary screening tool in women younger than 30 years. The high prevalence of high-risk human papillomavirus infections in the PALMS cohort (positivity rate of 20.2% in women younger than 30 years) indicates that the specificity of HPV testing in younger women in the PALMS cohort would be in the lower 80% range, confirming the limited clinical utility of pooled HPV testing in this age cohort. However, this study confirms previous findings that there is a substantial amount of CIN2+ in this age group. Almost 40% of the CIN2+ and CIN3+ cases were identified in women younger than age 30, although this age group represented only 25% of all PALMS participants.

A limitation of the study is that for logistical feasibility reasons dual-stained cytology testing was performed in a centralized laboratory. The screening of slides for the presence of dual-stained cells was performed by a group of contracted cytotechnologists who read study cases (approximately 10 per hour, equivalent to 80 slides per working day) besides their non–study-related routine Pap cytology work. The fact that for study purposes all slides were screened by two cytotechnologists before results were confirmed by pathologist review may overestimate the sensitivity of dual-stained cytology in this study. In contrast, the lack of a discussion of discrepant results between cytotechnologists and pathologists (as it would occur in daily routine practice) may have led to an unfavorable bias for dual-stained cytology testing.

It will be important to study in more detail the combination of dual-stained cytology and HPV testing in current Pap/HPV co-testing or upcoming HPV primary screening algorithms. The potential of dual-stained cytology as an effective triage of women 30 years or older with NILM/HPV+ co-testing results has been demonstrated recently. The vast majority of CIN2+ disease (>90%) was identified within the 25% of NILM/HPV+ women who were tested positive for the presence of dual-stained cells, allowing for the early identification of women who benefit most from immediate colposcopy (18). Furthermore, assessing the correlation between dual-stained cytology and HPV16/18 genotyping will be of interest to understand the potential association between individual genotypes and dual-stained cytology test outcomes. In a recent study evaluating p16/Ki-67 dual-stained cytology in a large US colposcopy referral population with rigorous disease verification, the test was shown to identify all HPV16-associated CIN3+ lesions among women 30 years or older, whereas sensitivity rates for non–HPV16-associated lesions in women younger than age 30 showed the lowest sensitivity level of 78% (22). Genotyping information is not yet available for the PALMS cohort, but it might allow the assessment of potential synergies between both genotyping and dual-stained cytology as triage components in screening with molecular HPV tests, in reflex testing of abnormal Pap cytology results, and/or in women younger than 30 years and screened with dual-stained cytology.

Longitudinal data will be needed to assess the risk of developing HGCIN after a negative dual-stained cytology result. Recent data from a multicenter randomized trial (New Technologies for Cervical Cancer, NTCC, in Italy) comparing HPV testing with Pap cytology and using p16 cytology to triage positive HPV results suggest that immediate follow-up can be avoided in HPV-positive p16 cytology-negative women, who may be safely managed with follow-up testing within 2–3 years (15,16). The high sensitivity levels of dual-stained cytology for the detection of prevalent HGCIN (especially CIN3+) shown in several cross-sectional studies may hint at a comparable negative predictive value for negative test results of the dual-stained cytology assay when used for triaging HPV-positive women, as compared with the p16 cytology assay used in the NTCC trial (15–22,27).

In summary, the results of this large prospective study show that a single test for the presence of cervical epithelial cells co-expressing the p16/Ki-67 biomarkers represents a novel approach to screen efficiently for HGCIN, compensating the sensitivity gap of Pap cytology testing while maintaining its specificity. Its performance characteristics suggest dual-stained cytology may be an attractive tool, especially in screening for precancerous lesions in younger women, where currently no other adjunctive or alternative technology to Pap cytology is available.

## Funding

This work was supported by Roche mtm laboratories who funded the study and was responsible for developing the protocol in collaboration with the coordinating and other lead investigators in the five European countries participating in the study, and for all aspects of the study conduct, including study assessments, data processing and management, statistical analysis, and reporting of results.

## Supplementary Material

Supplementary Data

## Notes

HI, CB, DS, HG, CA, JB, RD, TK, MvKD, HHN, LPT, SR, and RR contributed to the study design. CA, JB, RD, HG, and LPT acted as the national study coordinators in the five European countries that enrolled women to the study. DS, FA, JB, KD, and JH performed the reading of dual-stained cytology specimens. TK, SR, and RR did the primary data analysis. All authors reviewed the data, revised the manuscript, and the corresponding author had final responsibility to submit for publication. HI, DS, HG, CB, JB, RD, KD, HHN, LMP, and the institution of MS received compensation for occasional participation in advisory meetings and reimbursement of travel expenses. Compensation for laboratory services within the course of this study was paid to the institutions of HI, DS, HG, CB, JB, RD, KD, HHN, LMP, and MS. HG has received reimbursement of travel expenses from Dr. Kade Pharmaceuticals Co., Germany. The institution of HHN has received reimbursement for travel expenses from BD Diagnostics. HI is an occasional adviser to Hologic and GenProbe, and on the speakers’ bureaus of BD Diagnostics, GenProbe, Hologic, Qiagen, Roche Diagnostics, and Sanofi Pasteur. His institution has received grants or contracts by Abbott, Otto Bock, and Roche Diagnostics. JB has been on the speakers’ bureau of BD Diagnostics and Hologic and received travel expenses. His institution has received compensation for board membership from Hologic. MvKD was a member of the supervisory board, member of the speakers’ bureaus, and had a financial interest in the sponsor when the study was performed. RR and SR were employees of Roche mtm laboratories, and RR had a financial interest in the company when the study was performed. TK was a contract statistical consultant to Roche mtm laboratories. We thank all general practitioners, gynecologists, laboratory staff, and clinical research staff in the participating institutions for their contributions to this study. We are grateful to Jim Ranger-Moore, Tucson/Arizona, for critical review of the manuscript and helpful discussions.

## Appendix 1

### Major Collaborators

**Austria:** Sigrid Regauer. **Belgium:** Annie Vereecken, Christophe Depuydt, Ina Benoy, Shaira Sahebali. **France:** Olivier Aynaud; Jean-Jacques Baldauf; Jean-Marc Bohbot; Christine Dépardon; Bernard Huynh; Philippe Judlin; Gerlinde Lang-Avérous; Béatrice Marie; Guiseppe Pollini. **Germany:** Olaf Bettendorf, Christoph Börsch, Friederike Brinkmann-Smetanay; Christine Buhrmann; Anita Eich; Stephan Falk; Manfred Johnscher; Bodo Jordan, Petra Klement; Frieder Kommoss; Volkmar Küppers; Marcel Marquardt; Yvonne Mikulec; H.J. Pablo Roth; James Seabert; Torsten Schmidt; Martin Tenger; Marcus J. Trunk; Ulrich Zahn. **Italy:** Amedeo Lattanzi; Antonio Angelonel; Domenico Angelucci; Donatella Caraceni; Chiara Casadio; Lucia Ciccocioppo; Gino Coletti; Ada D’Amario; Gilda Di Paolo; Camillo Di Giulio; Carmine Fortunato; Vincenzo Maccallini; Carla Peluzzi; Massimo Pensieri; Maria Antonella Piccirilli; Giuseppe Pizzicannella; Bruna Ponte; Tatiana Reggi; Sandra Rosini; Solidea Saltarelli; Enrico Scala; Rosella Stampone; Vera Stornelli; Alfonso Tiberi; Maria Antonietta Venditti; Maria Ventura. **Spain**: Rosa Almirall; Ignacio Aranda; Victoria Batolome; Montse Cardona; Pluvio Coronado; Marta Del Pino; Vicente Furió Bacete; Pere Fuste; Mireya Jimeno; Belén Lloveras Rubio; José Antonio López Garcia-Asenjo; Gemma Mancebo; Juan Carlos Martinez Escoriza; Roser Nonell; Jaume Ordi Majà; Francisco Javier Segui Ivànez; Aureli Torné Blade; Jose Antonio Vidart Arragon.
