# Supplementary material for: Screening for Cervical Cancer Precursors With p16/Ki-67 Dual-Stained Cytology: Results of the PALMS Study
Source: J Natl Cancer Inst. 2013 Oct 4;105(20):1550–7. doi: 10.1093/jnci/djt235 (PMC3814411; doi:10.1093/jnci/djt235)
Supplement: Supplementary Data [file supp_105_20_1550__index.html]

Screening for Cervical Cancer Precursors With p16/Ki-67 Dual-Stained Cytology: Results of the PALMS Study — Screening for Cervical Cancer Precursors With p16/Ki-67 Dual-Stained Cytology: Results of the PALMS Study — Supplementary Data 

# Screening for Cervical Cancer Precursors With p16/Ki-67 Dual-Stained Cytology: Results of the PALMS Study

## Supplementary Data

Data files

**Files in this Data Supplement:**

- Supplementary Data - Supplementary Data
