# Supplementary material for: Screening for Cervical Cancer Precursors With p16/Ki-67 Dual-Stained Cytology: Results of the PALMS Study
Source: J Natl Cancer Inst. 2013 Oct 4;105(20):1550–7. doi: 10.1093/jnci/djt235 (PMC3814411; doi:10.1093/jnci/djt235)
Supplement: Supplementary Data [file supp_djt235_jnci_JNCI_13_0627_s01.docx]

**Supplementary Methods**

**p16/Ki-67 Dual-stained cytology and Interpretation**

Immunostaining of cervical cytology preparations for p16/Ki-67 was performed using the CINtec PLUS Kit (REF 9531, Roche mtm laboratories) according to the manufacturer's instructions. This kit is designed to perform a two-step immunocytochemical staining procedure of alcohol-fixed cervical cytology preparations. The kit contains a ready-to-use primary antibody cocktail comprising a mouse monoclonal antibody (clone E6H4) directed to human p16INK4a (p16) protein and a rabbit monoclonal antibody (clone 274-11 AC3) directed against human Ki-67 protein. Additional ready-to-use kit reagents used comprise (i) a polymer reagent conjugated to horseradish peroxidase (HRP) and goat anti-mouse fragment antigen-binding Fab′ antibody fragments, and (ii) a polymer reagent conjugated to alkaline phosphatase (AP) and goat anti-rabbit Fab′ antibody fragments. HRP-mediated conversion of 3,3′-diaminobenzidine (DAB) chromogen, and AP-mediated conversion of Fast Red chromogen lead to brown and red staining at the p16 and Ki-67 protein sites, respectively. After counterstaining by alcohol-free hematoxylin, a two-step mounting procedure was applied, using an aqueous mounting medium provided with the kit to prevent alcohol-based fading of the Fast Red signal, followed by a permanent mounting step.

Slides were evaluated for technical adequacy and interpretability based on minimum squamous cellularity criteria and occurrence of obscuring elements, as defined by *The Bethesda system. Terminology for reporting results of cervical cytology* (23), and immunostaining artefacts. Cases not meeting those criteria were excluded from the study.

Interpretation of p16/Ki-67 dual-stain cytology slides was performed by trained cytotechnologists who reviewed the slides for the presence of double-immunoreactive cells. The presence of one or more cervical epithelial cell(s) simultaneously showing brown cytoplasmic (p16) and red nuclear (Ki-67) staining defined a positive test result. Morphology interpretation was not considered in the interpretation of dual-stained cytology slides. Cases without any dual-stained cell identified by the cytotechnologists were called negative. Positive cases per cytotechnologist review were confirmed by additional pathologist review.

**Statistical methods**

In an ideal setting, determination of the performance characteristics of a diagnostic screening test will be performed in a representative sample of the appropriate population with a validated reference standard result available for every member of the sample. In practice, this can be difficult to achieve, as it may be unethical or impractical to perform invasive confirmation techniques in subjects who initially screen as negative for disease. In this study, three diagnostic tests (dual-stained cytology, Pap cytology, HPV) were utilized. Under the assumption that the rate of false negatives among patients who test negative by all three methods is negligibly small, absolute measures of diagnostic accuracy were calculated and reported.

A VOPT (verification of only positive tests) approach was also used. The VOPT design does not require any assumption about the true status of those subjects who are negative by all three diagnostic tests. Instead, it focuses on relative diagnostic measures, specifically the rTPF (ratio of true positive fractions) and rFPF (ratio of false positive fractions) for pairwise test comparisons. Consider as an example the derivation of the rTPF. The TPF, also known as sensitivity, is the probability that an individual with disease (D+) experiences a positive test result (T+). In other words, TPF = P(T+|D+). When comparing two diagnostic tests, the following 2x2 table demonstrates the joint results for diagnostic test outcomes, where T_1_ and T_2_ represent the two tests. Note that this table conditions on D+, i.e. all observations included in this table have the disease.

**Table: Joint test results for two diagnostic tests among subjects who have disease (D+).**

|  |  | Test 1 | |  |
| --- | --- | --- | --- | --- |
|  |  | T_1_+ | T_1_- |  |
| Test 2 | T_2_+ | a | b | a+b |
|  | T_2_- | c | d | c+d |
|  |  | a+c | b+d | a+b+c+d |

The true positive fractions can be calculated as the proportion of subjects testing positive by a given test over the total number of subjects with disease. Therefore,

TPF_1_ = (a+c) / (a+b+c+d) (1)

and

TPF_2_ = (a+b) / (a+b+c+d) (2)

If a representative sample is chosen from the population of interest and true disease status is verified in everyone then the estimates given in (1) and (2) will be valid. However, when disease verification is conducted only in subjects testing positive by at least one test, then by definition it is not conducted among those subjects appearing in the cell belonging to double-negative test results (lower right corner, depicted as ‘d’). The lack of verification may result in an incorrect joint frequency count for that cell, resulting in biased estimation of TPF values in (1) and (2).

This problem disappears when considering the relative performance of the two tests:

rTPF_12_ = TPF_1_ / TPF_2_ = [(a+c) / (a+b+c+d)] / [(a+b) / (a+b+c+d)] = (a+c) / (a+b) (3)

In deriving the ratio of the TPF estimates, the total number of diseased subjects (a+b+c+d) drops out of the equation, so any inappropriate inclusion of false negative subjects in cell ‘d’ of the table above can no longer exert a biasing effect. The same line of argument applies to the consideration of rFPF.

Two-sided P values are asymptotically equivalent to those obtained from McNemar’s test while also correcting for verification bias (24). Specifically, confidence intervals are constructed for estimates of rTPF and rFPF in the context of paired data, with null hypothesis tests that rTPF = 1 or, similarly, that rFPF = 1. The same variance estimates used to build those confidence intervals are also used to obtain P values using the standard normal distribution.

**Supplementary Figure 1**

**Sensitivity versus Specificity for CIN2+ (Reference Standard H&E corr)**

Receiver-operator characteristic graph is shown for Pap cytology (squares), Dual-stained cytology (circles), and HPV (triangle) for women aged 18-65 (grey fill), <30 (white fill), and ≥30 (black fill).

**Pap**

**Dual-stain**

**HPV**

**Supplementary Table 1: Sensitivity, specificity and predictive values of Pap cytology, p16/Ki-67 dual-stained cytology, and HPV testing in screening for CIN2+ and CIN3+ (Reference standard H&E corr)***

|  | **CIN2+** |  | **CIN3+** |  | **CIN2+** |  |
| --- | --- | --- | --- | --- | --- | --- |
|  | **Sensitivity**  **%**  (95% CI) | **Specificity**  **%**  (95% CI) | **Sensitivity**  **%**  (95% CI) | **Specificity**  **%**  (95% CI) | **PPV**  **%** | **NPV**  **%** |
| **Women aged 18-65 years (n=25,577; 205 CIN2+, 111 CIN3+)** | | | | | | |
| Pap cytology | **66.4**  (59.5-72.6) | **95.4**  (95.2-95.7) | **71.3**  (61.9-79.1) | **95.1**  (94.9-95.4) | **14.6** | **99.6** |
| Dual-stained cytology | **90.1**  (85.3-93.5) | **95.3**  (95.0-95.6) | **90.4**  (83.5-94.6) | **94.9**  (94.6-95.1) | **18.4** | **99.9** |
| **Women aged 18-29 years (n=6,372; 82 CIN2+, 41 CIN3+)** | | | | | | |
| Pap cytology | **67.7**  (56.3-77.4) | **92.8**  (92.1-93.4) | **74.4**  (58.2-85.9) | **92.2**  (91.5-92.9) | **16.0** | **99.3** |
| Dual-stained cytology | **93.3**  (85.7-97.0) | **92.3**  (91.6-93.0) | **93.2**  (80.8-97.8) | **91.4**  (90.7-92.1) | **19.7** | **99.9** |
| **Women aged 30-65 years (n=19,205; 123 CIN2+, 70 CIN3+)** | | | | | | |
| Pap cytology | **64.9**  (56.0-72.9) | **96.3**  (96.0-96.6) | **69.1**  (57.1-78.9) | **96.1**  (95.8-96.4) | **13.7** | **99.7** |
| Dual-stained cytology | **87.8**  (80.8-92.5) | **96.3**  (96.0-96.6) | **88.5**  (78.7-94.1) | **96.0**  (95.7-96.3) | **17.6** | **99.9** |
| HPV | **95.6**  (89.0-98.3) | **93.1**  (92.7-93.5) | **100.0**  (94.9-100.0) | **92.8**  (92.4-93.2) | **10.5** | **>99.9** |

***** CIN2+ (CIN3+), cervical intraepithelial neoplasia grade 2 (3) or worse; HPV, human papillomavirus; 95% CI, 95% confidence intervals; PPV, positive predictive value; NPV, negative predictive value. Data are provided using the histological diagnoses from a central pathology review as the gold standard where the initial diagnosis on tissue biopsies was not supported by immunohistochemical staining pattern and thus underwent a second adjudication review (Reference standard H&E *corr*)

**Supplementary Table 2: Relative performance characteristics of Pap cytology, p16/Ki-67 dual-stained cytology, and HPV testing for detection of CIN2+ and CIN3+ (Reference standard H&E corr)***

|  | **CIN2+** |  |  |  | **CIN3+** |  |  |  |
| --- | --- | --- | --- | --- | --- | --- | --- | --- |
|  | **Relative Sensitivity rTPF**  (95% CI) | **P value**  **rTPF** | **Relative Specificity**  **rFPF**  (95% CI) | **P value**  **rFPF** | **Relative Sensitivity**  **rTPF**  (95% CI) | **P value**  **rTPF** | **Relative Specificity**  **rFPF**  (95% CI) | **P value**  **rFPF** |
| **Women aged 18-65 years (n=25,577; 205 CIN2+, 111 CIN3+)** | | | | | | |  |  |
| Dual-stained cytology vs. Pap cytology | **1.358**  (1.220-1.513) | **< .001** | **1.030**  (0.963-1.101) | **.39** | **1.268**  (1.111-1.447) | **< .001** | **1.060**  (0.996-1.128) | **.06** |
| **Women aged 18-29 years (n=6,372; 82 CIN2+, 41 CIN3+)** | | | | | | |  |  |
| Dual-stained cytology vs. Pap cytology | **1.377**  (1.160-1.633) | **< .001** | **1.066**  (0.961-1.181) | **.23** | **1.252**  (1.009-1.553) | **.04** | **1.102**  (1.004-1.211) | **.04** |
| **Women aged 30-65 years (n=19,205; 123 CIN2+, 70 CIN3+)** | | | | | | |  |  |
| Dual-stained cytology vs. Pap cytology | **1.352**  (1.176-1.555) | **< .001** | **1.006**  (0.921-1.098) | **.90** | **1.281**  (1.083-1.516) | **.004** | **1.033**  (0.951-1.122) | **.45** |
| Dual-stained cytology vs. HPV | **0.909**  (0.834-0.991) | **.03** | **0.542**  (0.504-0.583) | **< .001** | **0.876**  (0.800-0.959) | **.004** | **0.561**  (0.524-0.601) | **< .001** |

***** CIN2+ (CIN3+), cervical intraepithelial neoplasia grade 2 (3) or worse; HPV, human papillomavirus; 95% CI, 95% confidence intervals; rTPF, ratio of true positive fractions (relative sensitivity); rFPF, ratio of false positive fractions (relative 1-specificity). Data are provided using the histological diagnoses from a central pathology review as the gold standard where the initial diagnosis on tissue biopsies was not supported by immunohistochemical staining pattern and thus underwent a second adjudication review (Reference standard H&E *corr*). Two-sided bias-corrected McNemar P values are reported

**Supplementary Table 3: Absolute and relative performance characteristics of Pap cytology and p16/Ki-67 dual-stained cytology for detection of CIN2+ per Pap cytology method (Reference standard H&E corr)***

|  | **Dual-stained cytology** | | **Pap cytology** | | **Dual-stained vs. Pap cytology** | |
| --- | --- | --- | --- | --- | --- | --- |
|  | **Sensitivity**  **%**  (95% CI) | **Specificity %**  (95% CI) | **Sensitivity**  **%**  (95% CI) | **Specificity %**  (95% CI) | **Relative Sensitivity**  **rTPF**  (95% CI) | **Relative Specificity**  **rFPF**  (95% CI) |
| All | **90.1**  (85.3-93.5) | **95.3**  (95.0-95.6) | **66.4**  (59.5-72.6) | **95.4**  (95.2-95.7) | **1.358**  (1.220-1.513) | **1.030**  (0.963-1.101) |
| Conventional | **87.2**  (78.4-92.8) | **95.8**  (95.4-96.2) | **63.6**  (52.9-73.1) | **97.5**  (97.2-97.8) | **1.371**  (1.143-1.645) | **1.705**  (1.492-1.947) |
| Surepath | **88.1**  (77.0-94.2) | **95.1**  (94.6-95.6) | **54.8**  (41.4-67.5) | **93.0**  (92.4-93.6) | **1.607**  (1.240-2.082) | **0.699**  (0.621-0.786) |
| ThinPrep | **95.6**  (87.1-98.6) | **95.0**  (94.4-95.4) | **80.2**  (68.5-88.3) | **95.1**  (94.6-95.6) | **1.191**  (1.046-1.356) | **1.032**  (0.926-1.151) |

***** CIN2+, cervical intraepithelial neoplasia grade 2 or worse; 95% CI, 95% confidence intervals; rTPF, ratio of true positive fractions (relative sensitivity); rFPF, ratio of false positive fractions (relative 1-specificity)

**Supplementary Table 4: Relative performance characteristics of Pap cytology, p16/Ki-67 dual-stained cytology, and HPV testing for detection of CIN2+ and CIN3+; ThinPrep cytology cases only; Reference standard H&E (black) and H&E *corr* (blue) ***

|  | **CIN2+** |  |  |  | **CIN3+** |  |  |  |
| --- | --- | --- | --- | --- | --- | --- | --- | --- |
|  | **Relative Sensitivity**  **rTPF** (95% CI) | **P value**  **rTPF** | **Relative Specificity**  **rFPF** (95% CI) | **P value**  **rFPF** | **Relative Sensitivity**  **rTPF** (95% CI) | **P value**  **rTPF** | **Relative Specificity**  **rFPF** (95% CI) | **P value**  **rFPF** |
| **Women aged 18-65 years (n=8,708; 58 (67) CIN2+, 36 (41) CIN3+)** | | | | | | |  |  |
| Dual-stained cytology vs. Pap cytology | **1.079** (0.941-1.237)  **1.191** (1.046-1.356) | **.28**  **.01** | **1.053** (0.947-1.170)  **1.032 (0.926-1.151)** | **.34**  **.56** | **1.082** (0.919-1.274)  **1.180** (0.985-1.413) | **.35**  **.07** | **1.054** (0.954-1.165)  **1.044** (0.944-1.155) | **.30**  **.40** |
| **Women aged 18-29 years (n=1,938; 26 (34) CIN2+, 13 (16) CIN3+)** | | | | | | |  |  |
| Dual-stained cytology vs. Pap cytology | **1.006** (0.878-1.152)  **1.226** (1.033-1.454) | **.94**  **.02** | **1.182** (1.003-1.393)  **1.129** (0.950-1.343) | **.05**  **.17** | **1.109** (0.908-1.354)  **1.302** (0.977-1.735) | **.31**  **.07** | **1.154** (0.994-1.339)  **1.134** (0.975-1.318) | **.06**  **.10** |
| **Women aged 30-65 years (n=6,770; 32 (33) CIN2+, 23 (25) CIN3+)** | | | | | | |  |  |
| Dual-stained cytology vs. Pap cytology | **1.141** (0.909-1.432)  **1.171** (0.955-1.435) | **.26**  **.13** | **0.983** (0.856-1.128)  **0.978** (0.851-1.124) | **.80**  **.75** | **1.067** (0.847-1.343)  **1.116** (0.883-1.410) | **.58**  **.36** | **0.994** (0.869-1.136)  **0.989** (0.864-1.131) | **.93**  **.87** |
| Dual-stained cytology vs. HPV | **0.941** (0.813-0.990)  **0.944** (0.822-1.084) | **.41**  **.41** | **0.513** (0.457-0.575)  **0.511** (0.455-0.573) | **< .001**  **< .001** | **0.867** (0.739-1.018)  **0.877** (0.757-1.017) | **.08**  **.08** | **0.524** (0.469-0.586)  **0.522** (0.466-0.584) | **< .001**  **< .001** |

***** CIN2+ (CIN3+), cervical intraepithelial neoplasia grade 2 (3) or worse; HPV, human papillomavirus; 95% CI, 95% confidence intervals; rTPF, ratio of true positive fractions (relative sensitivity); rFPF, ratio of false positive fractions (relative 1-specificity). Two-sided bias-corrected McNemar P values are reported

**Supplementary Table 5: Relative performance characteristics of Pap cytology, p16/Ki-67 dual-stained cytology, and HPV testing for detection of CIN2+ and CIN3+; SurePath cytology cases only; Reference standard H&E (black) and H&E *corr* (blue) ***

|  | **CIN2+** |  |  |  | **CIN3+** |  |  |  |
| --- | --- | --- | --- | --- | --- | --- | --- | --- |
|  | **Relative Sensitivity**  **rTPF** (95% CI) | **P value**  **rTPF** | **Relative Specificity**  **rFPF** (95% CI) | **P value**  **rFPF** | **Relative Sensitivity**  **rTPF** (95% CI) | **P value**  **rTPF** | **Relative Specificity**  **rFPF** (95% CI) | **P value**  **rFPF** |
| **Women aged 18-65 years (n=7,096; 47 (56) CIN2+, 23 (29) CIN3+)** | | | | | | |  |  |
| Dual-stained cytology vs. Pap cytology | **1.435** (1.071-1.921)  **1.607** (1.240-2.082) | **.02**  **< .001** | **0.722** (0.644-0.810)  **0.699 (0.621-0.786)** | **< .001**  **< .001** | **1.487** (1.015-2.179)  **1.615** (1.160-2.248) | **.04**  **.004** | **0.747** (0.670-0.833)  **0.735** (0.659-0.821) | **< .001**  **< .001** |
| **Women aged 18-29 years (n=2,021; 18 (20) CIN2+, 13 (13) CIN3+)** | | | | | | |  |  |
| Dual-stained cytology vs. Pap cytology | **1.907** (1.064-3.418)  **1.851** (1.133-3.025) | **.03**  **.01** | **0.612** (0.509-0.736)  **0.601** (0.497-0.726) | **< .001**  **< .001** | **1.355** (0.776-2.365)  **1.461** (0.881-2.421) | **.29**  **.14** | **0.655** (0.549-0.780)  **0.648** (0.543-0.774) | **< .001**  **< .001** |
| **Women aged 30-65 years (n=5,075; 29 (36) CIN2+, 10 (16) CIN3+)** | | | | | | |  |  |
| Dual-stained cytology vs. Pap cytology | **1.286** (0.937-1.766)  **1.537** (1.136-2.078) | **.12**  **.005** | **0.789** (0.680-0.916)  **0.760** (0.652-0.886) | **.002**  **< .001** | **1.843** (1.038-3.271)  **1.843** (1.156-2.938) | **.04**  **.01** | **0.805** (0.700-0.925)  **0.791** (0.686-0.912) | **.002**  **.001** |
| Dual-stained cytology vs. HPV | **0.804** (0.667-0.970)  **0.881** (0.738-1.051) | **.02**  **.16** | **0.753** (0.659-0.861)  **0.741** (0.645-0.850) | **< .001**  **< .001** | **1.000** (1.000-1.000)  **0.928** (0.801-1.074) | **1.00**  **.31** | **0.750** (0.662-0.849)  **0.748** (0.659-0.850) | **< .001**  **< .001** |

***** CIN2+ (CIN3+), cervical intraepithelial neoplasia grade 2 (3) or worse; HPV, human papillomavirus; 95% CI, 95% confidence intervals; rTPF, ratio of true positive fractions (relative sensitivity); rFPF, ratio of false positive fractions (relative 1-specificity). Two-sided bias-corrected McNemar P values are reported

**Supplementary Table 6: Relative performance characteristics of Pap cytology, p16/Ki-67 dual-stained cytology, and HPV testing for detection of CIN2+ and CIN3+; Conventional cytology cases only; Reference standard H&E (black) and H&E *corr* (blue) ***

|  | **CIN2+** |  |  |  | **CIN3+** |  |  |  |
| --- | --- | --- | --- | --- | --- | --- | --- | --- |
|  | **Relative Sensitivity**  **rTPF** (95% CI) | **P value**  **rTPF** | **Relative Specificity**  **rFPF** (95% CI) | **P value**  **rFPF** | **Relative Sensitivity**  **rTPF** (95% CI) | **P value**  **rTPF** | **Relative Specificity**  **rFPF** (95% CI) | **P value**  **rFPF** |
| **Women aged 18-65 years (n=9,773; 76 (82) CIN2+, 41 (41) CIN3+)** | | | | | | |  |  |
| Dual-stained cytology vs. Pap cytology | **1.338** (1.107-1.618)  **1.371** (1.143-1.645) | **.003**  **< .001** | **1.707** (1.498-1.944)  **1.705 (1.492-1.947)** | **< .001**  **< .001** | **1.146** (0.913-1.437)  **1.173** (0.950-1.447) | **.24**  **.14** | **1.699** (1.509-1.913)  **1.697** (1.506-1.912) | **< .001**  **< .001** |
| **Women aged 18-29 years (n=2,413; 26 (28) CIN2+, 11 (12) CIN3+)** | | | | | | |  |  |
| Dual-stained cytology vs. Pap cytology | **1.262** (0.931-1.710)  **1.369** (0.995-1.885) | **.13**  **.05** | **1.896** (1.553-2.314)  **1.860** (1.525-2.268) | **< .001**  **< .001** | **0.921** (0.602-1.407)  **1.108** (0.751-1.633) | **.70**  **.61** | **1.861** (1.558-2.222)  **1.836** (1.536-2.194) | **< .001**  **< .001** |
| **Women aged 30-65 years (n=7,360; 50 (54) CIN2+, 30 (29) CIN3+)** | | | | | | |  |  |
| Dual-stained cytology vs. Pap cytology | **1.393** (1.090-1.779)  **1.379** (1.103-1.724) | **.008**  **.005** | **1.570** (1.321-1.866)  **1.582** (1.324-1.891) | **< .001**  **< .001** | **1.277** (0.969-1.684)  **1.212** (0.939-1.565) | **.08**  **.14** | **1.573** (1.342-1.845)  **1.588** (1.352-1.863) | **< .001**  **< .001** |
| Dual-stained cytology vs. HPV | **0.913** (0.777-1.073)  **0.899** (0.785-1.029) | **.27**  **.12** | **0.458** (0.403-0.520)  **0.452** (0.397-0.516) | **< .001**  **< .001** | **0.886** (0.734-1.070)  **0.847** (0.721-0.995) | **.21**  **.04** | **0.481** (0.427-0.542)  **0.483** (0.429-0.544) | **< .001**  **< .001** |

***** CIN2+ (CIN3+), cervical intraepithelial neoplasia grade 2 (3) or worse; HPV, human papillomavirus; 95% CI, 95% confidence intervals; rTPF, ratio of true positive fractions (relative sensitivity); rFPF, ratio of false positive fractions (relative 1-specificity). Two-sided bias-corrected McNemar P values are reported
